# Developmental Olfactory Dysfunction and Abnormal Odor Memory in Immune-Challenged *Disc1^+/−^* Mice

**DOI:** 10.1523/JNEUROSCI.1007-24.2025

**Published:** 2025-05-30

**Authors:** Fiona Parbst, Johanna K. Kostka, Anne Günther, Yu-Nan Chen, Ileana L. Hanganu-Opatz, Sebastian H. Bitzenhofer

**Affiliations:** ^1^Institute of Developmental Neurophysiology, Center for Molecular Neurobiology Hamburg, Hamburg Center of Neuroscience, University Medical Center Hamburg-Eppendorf, Hamburg 20251, Germany; ^2^Neural Circuit Physiology, Center for Molecular Neurobiology Hamburg, University Medical Center Hamburg-Eppendorf, Hamburg 20251, Germany

**Keywords:** development, Disc1, hippocampus, neuropsychiatric, olfaction, prefrontal cortex

## Abstract

Neuronal activity in the olfactory bulb (OB) drives coordinated activity in the hippocampal–prefrontal network during early development. Inhibiting OB output in neonatal mice disrupts functional development of the hippocampal formation as well as cognitive abilities. These impairments manifest early in life and resemble dysfunctions of the hippocampus and the prefrontal cortex that have been linked to neuropsychiatric disorders. Thus, we investigated OB activity during early development in a disease mouse model and asked whether activity disruptions might contribute to the dysfunctional development of the hippocampal–prefrontal network. We addressed this question by combining in vivo electrophysiology with behavioral assessment of immune-challenged *Disc1^+/−^* mice of both sexes that mimic the dual genetic–environmental etiology of neuropsychiatric disorders. In wild-type mice, we found high DISC1 expression levels in OB projection neurons during development. Furthermore, neuronal and network activity in the OB and the drive from the bulb to the hippocampal–prefrontal network were reduced in immune-challenged *Disc1^+/−^* mice during early development. This early deficit did not affect odor-evoked activity and odor perception but resulted in impaired long-term odor memory. We propose that reduced spontaneous activity in the developing OB might contribute to altered maturation of the hippocampal–prefrontal network, leading to memory impairment in immune-challenged *Disc1^+/−^* mice.

## Significance Statement

Cognitive deficits in neuropsychiatric disorders result from dysfunctional activity within hippocampal–prefrontal networks manifesting early in life. Input from the olfactory system is critical for the maturation of coordinated activity in hippocampal–prefrontal networks and cognitive abilities in mice. Thus, we hypothesized that early activity in the olfactory system might be altered in a mouse model of neuropsychiatric disorders. Indeed, we found reduced activity in the olfactory bulb (OB) in this mouse model resulting in impaired interaction with the hippocampus and prefrontal cortex. Surprisingly, this impairment did not affect odor-evoked activity and odor perception but resulted in deficient long-term odor memory. These results indicate that reduced developmental activity in the OB might contribute to the etiology of neuropsychiatric disorders.

## Introduction

Functional maturation of the central nervous system is shaped by neuronal activity (Katz and Shatz, 1996; Kirkby et al., 2013; Kirischuk et al., 2017; Hanganu-Opatz et al., 2021). This activity can be generated within local networks or driven by the sensory periphery. In rodents, developmental activity in the visual cortex, generated locally or by retinal waves, is critical for network refinement (Hanganu et al., 2006; Ackman et al., 2012). Similarly, spontaneous cochlear activity supports maturation of the auditory cortex (Clause et al., 2014; Kersbergen et al., 2022). Higher association areas, such as the prefrontal cortex (PFC), are also shaped by early neuronal activity. Disruptions during early postnatal development can cause long-lasting dysfunctions in neuronal circuits and cognitive abilities due to excitation–inhibition imbalance (Bitzenhofer et al., 2021; Medendorp et al., 2021).

However, what is driving early activity in the PFC is less clear. During development, inputs from the mediodorsal thalamus increase activity in the PFC and their inhibition impairs prefrontal maturation (Benoit et al., 2022). Monosynaptic projections from the intermediate/ventral hippocampus provide another excitatory drive for developmental prefrontal activity (Brockmann et al., 2011; Ahlbeck et al., 2018). A prominent candidate for the drive of developmental activity in these areas is the olfactory system. From birth on, mice rely on olfaction to find the teats of their dam for feeding (Logan et al., 2012). In line with this vital function, mitral and tufted cells, the projection neurons of the olfactory bulb (OB), develop prenatally, and their downstream connectivity is largely established at birth (Walz et al., 2006; Hirata et al., 2019). Coordinated neuronal activity in the OB emerges early and is more prominent than in other brain regions during the early postnatal period (Fletcher et al., 2005; Gretenkord et al., 2019). Even without direct connections from the OB to the hippocampus or PFC, strong projections from OB to the piriform cortex and lateral entorhinal cortex provide a short pathway by which the olfactory system can influence the hippocampal–prefrontal network (Igarashi et al., 2012; Witter et al., 2017; Kostka and Bitzenhofer, 2022a). Supporting this, we previously showed that rhythmic activity in the OB entrains the entorhinal cortex, hippocampus, and PFC during early postnatal development (Gretenkord et al., 2019; Kostka and Hanganu-Opatz, 2023). Thus, activity in the olfactory system may serve a role for the maturation of the hippocampal–prefrontal network, similar to retinal and cochlear activity for the visual and auditory system.

Supporting this, we recently demonstrated that transient inhibition of OB outputs during postnatal development in mice reduces coordinated activity in the hippocampal formation and impairs cognitive abilities later in life (Chen et al., 2023). These findings resemble the developmental deficits observed in mouse models of neuropsychiatric disorders (Chini and Hanganu-Opatz, 2021; Günther and Hanganu-Opatz, 2022). Accumulating evidence suggests an association of olfactory impairment with neuropsychiatric disorders. Inflammation of the olfactory epithelium, reduced OB volume, and deficits in odor perception have been reported for schizophrenia, psychosis, and depression (Turetsky et al., 2003; Nguyen et al., 2010; Hasegawa et al., 2022; Herrmann et al., 2023; Yang et al., 2024). However, it remains unknown how alterations in the olfactory system might contribute to hippocampal–prefrontal network deficits associated with these disorders (Uhlhaas and Singer, 2010; Chini and Hanganu-Opatz, 2021).

Here, we investigated how the olfactory system interacts with the hippocampal–prefrontal network during early postnatal development in a mouse model of neuropsychiatric disorders. We employed immune-challenged *Disc1^+/−^* mice, a dual-hit mouse model which combines two well-established models for neuropsychiatric disorders: a heterozygous mutation in the gene disrupted-in-schizophrenia 1 (*Disc1^+/−^*) resulting in a truncated DISC1 protein (Kvajo et al., 2008; Brandon and Sawa, 2011) and maternal immune activation by the viral RNA mimetic polyinosinic–polycytidylic acid [poly(I:C); Meyer and Feldon, 2012]. This gene–environment (GE) model mimics the etiology of neuropsychiatric disorders and shows impaired developmental activity in the hippocampal–prefrontal network and deficits in associated cognitive tasks (Hartung et al., 2016; Chini et al., 2020; Xu et al., 2021b).

## Materials and Methods

### Ethical approval

All experiments were performed in compliance with German laws and guidelines of the European Union for the use of animals in research (EU Directive 2010/63/EU) and were approved by the local authorities (Behörde für Justiz und Verbraucherschutz Hamburg).

### Animals

Timed-pregnant mice from the University Medical Center Hamburg-Eppendorf animal facility were housed individually in a 12/12 h light/dark cycle and had access to water and food *ad libitum*. The day of vaginal plug detection was defined as Gestational Day 0.5, and the day of birth was defined as Postnatal Day (P)0. Experiments were performed on pups of both sexes during neonatal development (i.e., P8–11).

Pregnant *Disc1* mice (B6.129S6-Disc1^tm1Kara^) carrying a mutation resulting in a truncated transcript on a C57BL6/J background (Kvajo et al., 2008) received viral RNA mimetic poly(I:C) (25 mg/kg) injected intraperitoneally at Gestational Day 9.5 to induce maternal immune activation. Immune-challenged *Disc1^+/−^* mice (referred to as GE) combine genetic and environmental risk factors in the pathogenesis of neuropsychiatric disorders. Offspring of C57BL/6J mice were used as wild-type control animals (referred to as WT). Tbet-cre mice [B6; CBA-Tg (Tbx21-cre) 1Dlc/J, JAX#024507] were used for expression of Designer Receptors Activated Only by Designer Drugs (DREADDs) in mitral/tufted cells.

### Histology

P9–10 mice were anesthetized with ketamine (12 mg/ml, aniMedica International)/xylazine (1.6 mg/ml, WDT) in 0.9% NaCl (15 μl/g body weight, i.p.) and transcardially perfused with 4% paraformaldehyde (Histofix, Carl Roth). Brains were removed and postfixed in 4% paraformaldehyde for 24 h. Brains were sectioned coronally with a vibratome at 100 μm for immunostaining.

#### Immunostaining

Free-floating slices were permeabilized in 0.3% (v/v) H_2_O_2_ (in PBS) for 30 min at room temperature. Subsequently, unspecific binding sites were blocked in PBS containing 0.8% (v/v) Triton X-100 (Sigma-Aldrich), 5% (v/v) normal goat serum, and 5% (v/v) normal donkey serum (Jackson ImmunoResearch Laboratories) for 1 h at room temperature. Slices were incubated with primary antibodies in 0.8% (v/v) Triton X-100, 1% (v/v) normal goat serum, and 1% (v/v) normal donkey serum (in PBS) for 3 d at 4°C. Primary antibodies for DISC1 (1:300, rb-α-Disc1, Invitrogen, 40-6800) and NeuN (1:500, ms-α-NeuN, Millipore Sigma, MAB377) were used for colabeling. Subsequently, slices were washed in PBS and incubated with secondary antibodies (gt-α-rb-DyLight633, 1:1,000, Thermo Fisher Scientific, A32731; gt-α-ms-A488, 1:1,000, Thermo Fisher Scientific, A11029) for 3 h at room temperature in 0.8% (v/v) Triton X-100, 1% (v/v) normal goat serum, and 1% (v/v) normal donkey serum (in PBS). Slices were washed in PBS and transferred to glass slides, before being covered with VECTASHIELD (VectorLabs). Images of immunostainings were acquired with a confocal microscope (FV1000, Olympus), using a 20× objective (2× zoom, *z*-stacks of 11 images 1.5 µm). Acquisition settings were kept constant for all images. Images were processed and analyzed with ImageJ. *Z*-projections of the average intensity were applied to reduce image stacks to 2D images. Average pixel intensity of immunostainings against Disc1 was quantified in rectangles of 100 × 300 μm oriented with the shorter edge parallel to the OB surface, thereby spanning across the glomerular layer, external plexiform layer (EPL), mitral cell layer (MCL), and granule cell layer (GCL).

#### Retrograde tracing

P5 mice were injected with the retrograde tracer CTB555 (200 nl at 100 nl/min, cholera toxin subunit B, Alexa Fluor 455 conjugate) into the piriform cortex under isoflurane anesthesia (induction, 5%; maintenance, 2%). Pups were transcardially perfused, and brains were removed for immunostaining at P10.

### Surgical procedure for electrophysiology

For in vivo electrophysiological recordings, P8–10 mice underwent surgery under isoflurane anesthesia (induction, 5%; maintenance, 2%). The skin above the skull was removed, and local anesthetic (0.5% bupivacaine/1% lidocaine) was applied on the neck muscles. Two plastic bars were fixed on the nasal and occipital bones with dental cement. Craniotomies of ∼0.5 mm diameter were performed above the right OB (0.5–0.8 mm anterior to the frontonasal suture, 0.5 mm lateral to the internasal suture), the CA1 subdivision of the intermediate hippocampus (3.5 mm posterior to the bregma, 3.5 mm lateral to the midline), and the medial part of the PFC (0.5 mm anterior to the bregma; 0.1–0.5 mm lateral to the midline). Throughout surgery, recovery, and recording, mice were kept on a heating blanket at 37°C.

### Electrophysiological recordings

Extracellular recordings were performed simultaneously from the ventral OB, hippocampal CA1, and PFC in nonanesthetized P8–10 mice. For this, one-shank silicon probes (NeuroNexus) with 16 recording sites (50 µm intersite spacing) were inserted into OB (0.5–1.8 mm deep, angle 0°), CA1 (1.3–1.9 mm deep, angle 20°), and PFC (1.8–2.1 mm deep, angle 0°). Before insertion, the electrodes were covered with DiI (1,1′-dioctadecyl-3,3,3′,3′-tetramethylindocarbocyanine perchlorate, Molecular Probes) for confirmation of electrode position postmortem. A silver wire was inserted into the cerebellum and served as ground and reference electrode. A recovery period of 20 min after the insertion of electrodes was provided before data acquisition. Extracellular signals were bandpass filtered (0.1–9,000 Hz) and digitized (32 kHz) with a multichannel extracellular amplifier (Digital Lynx SX; NeuraLynx) and the Cheetah acquisition software (NeuraLynx).

After recordings, mice were deeply anesthetized with ketamine (12 mg/ml)/xylazine (1.6 mg/ml) in 0.9% NaCl solution (15 µl/g body weight, i.p.) and transcardially perfused with Histofix (Carl Roth) containing 4% paraformaldehyde for subsequent identification of electrode positions in coronal slices.

### Olfactory stimulation

A custom-made, Arduino-controlled olfactometer with a constant stream of clean air (0.9 L/min) to the nose was used to present odors to the animals. Odors were presented for 2 s triggered by the respiration cycle of mice to ensure constant odor concentration at the first odor inhalation. Two different odors (ethyl butyrate and isoamyl acetate, 1% in mineral oil) were delivered in a randomized order for 40 repetitions each.

### DREADD-mediated inhibition of OB outputs

Tbet-cre mouse pups were anesthetized with isoflurane (induction, 5%; maintenance, 2%) and fixed into a stereotaxic apparatus at P1. AAV9-EF1a-DIO-hM4Di-mCherry (Plasmid #50461, Addgene) at a titer of 1 × 10^13^ vg/ml was injected bilaterally into the OB (200 nl per side at 100 nl/min) using a microinjection pump [MICRO4, World Precision Ibstruments (WPI)]. After injections, pups were maintained on a heating blanket until full recovery and returned to the home cage. At P9–10, electrophysiological recordings were performed as described above. For chemogenetic inhibition of vesicle release of mitral/tufted cell axon terminals, Compound 21 (C21; Hello Bio, 3 mg/kg) was injected intraperitoneally during electrophysiological recordings. Expression of mCherry was confirmed postmortem.

### shRNA-mediated knockdown of DISC1

P1 immune-challenged C57BL6/J mouse pups were injected bilaterally into the OB (200 nl per side at 100 nl/min at titer of 7 × 10^13^ vg/ml) using a microinjection pump (MICRO4, WPI) with AAV9-hSyn-EGFP_H1-shRNA encoding short-hairpin RNA (shRNA) against *Disc1* (5-GGCAAACACTGTGAAGTGC-3) to induce spatially restricted, selective knockdown of DISC1 expression in the OB during early postnatal development. Control mice were injected with a virus encoding for shRNA with a scrambled target sequence (5-ATCTCGCTTGGGCGAGAGT-3). Both shRNAs were previously used for developmental DISC1 knockdown in the HP (Xu et al., 2021a) and PFC (Xu et al., 2019). At P8–10, electrophysiological recordings were performed as described above. Expression of EGFP was confirmed postmortem.

### Analysis of electrophysiological data

Electrophysiological data were analyzed with custom-written algorithms in MATLAB R2021a environment. For local field potential (LFP) analysis, data were bandpass filtered (1–100 Hz) using a phase preserving third-order Butterworth filter. For LFP data recorded in the OB, the recording site centered in the EPL was used, whereas, for the analysis of spiking activity, recording sites in the MCL or GCL were considered. For the analysis of hippocampal LFP, a recording site located in CA1 below the pyramidal layer was selected, while for the analysis of spiking activity, all recording sites located in CA1 were used. For LFP analysis in the PFC, a recording site centered in the prelimbic region was considered, and spiking activity from all recording sites was included.

#### Power spectral density

Power spectral density was calculated using Welch's method with nonoverlapping windows of 2 s for spontaneous activity or for a 2 s window for odor stimulation. Time–frequency power plots were calculated with a continuous wavelet transform (Morlet wavelet). Frequency bands for statistical comparisons were defined as respiration rhythm (RR; 2–4 Hz), theta (4–12 Hz), and beta (12–30 Hz).

#### Frequency-resolved amplitude correlation

LFP from OB, CA1, and PFC was bandpass filtered in frequency bins of 2 Hz from 1 to 30 Hz and Hilbert transformed to extract the absolute amplitude. Subsequently, pairwise Pearson's correlation coefficients of frequency-resolved envelopes were calculated for OB, CA1, and PFC.

#### Generalized partial directed coherence (gPDC)

gPDC was calculated in the frequency domain to investigate the directional interaction between areas. This linear Granger causality measure is based on the decomposition of multivariate partial coherence computed from multivariate autoregressive models. LFP signals of 1 s length were denoised using the MATLAB wavelet toolbox (ddencmp.m and wdencmp.m) before gPDC was calculated with a previously described algorithm (Baccalá and Sameshima, 2001).

#### Phase–amplitude coupling (PAC)

Cross-frequency coupling was calculated between the phase of the slow frequency in OB and the amplitude at fast frequencies (12–80 Hz) in CA1 and PFC according to a previously described algorithm (Tort et al., 2010). Bandpass filtered LFP was Hilbert transformed to extract the phase and amplitude. The amplitude of the 12–50 Hz filtered LFP in CA1 and PFC was determined at each phase of the filtered OB signal. PAC matrices were *z*-scored, and the average was calculated for RR (2–4 Hz) phase to higher frequencies (13–50 Hz) coupling.

#### Spiking analysis

Single units were automatically detected and clustered using the python-based software klusta (Rossant et al., 2016) and manually curated using phy (https://github.com/cortex-lab/phy). For OB recordings, units detected around the channel (±1 channels) where the RR reverses in polarity were considered for MCL spiking activity, whereas units in channels (>3 channels central from the MCL) were considered for GCL spiking activity.

Experimenters were not blind for the treatment group in electrophysiological experiments. To reduce experimenter bias, analyses were performed using automated analysis methods (MATLAB scripts for general analysis, klusta for spike sorting). The manual parts of the analyses were performed before group results were compared.

### Behavior

#### Neonatal odor detection

The suppression of ultrasonic vocalizations (USVs) of neonatal mice when exposed to the odor citral was used to test for neonatal odor detection. For each test, a P9 mouse was removed from the home cage, placed in a soundproof test box, and allowed to accommodate for 120 s. Pressurized air was pumped through the box at a rate of 2 L/min. USVs were recorded with an ultrasonic microphone (Avisoft-UltraSoundGate, Avisoft Bioacoustics) at a sampling rate of 250 kHz for 90 s with clean air, 60 s with citral, and 60 s of clean air, followed by a 60 s break. Each test consisted of three consecutive trials with increasing concentration of citral at 10^−4^, 10^−2^, and 1% diluted in mineral oil. USVs from 25 to 125 kHz were detected using DeepSqueak (Coffey et al., 2019).

#### Neonatal odor learning

In a separate cohort of mice, neonatal odor learning of P10 mice was assessed with a modified version of a previously established protocol (Armstrong et al., 2006). For one-trial associative odor learning, the dam was removed from the home cage for 2 h before the test odor was applied to the teats of the dam with a saturated cotton swab, and it was placed back to the home cage for 1 h. Isoamyl acetate and ethyl butyrate (1% in mineral oil, v/v) were used randomly as test and control odors per litter. The dam was removed again for 2 h before the pups were tested in an odor–place preference test. The test arena consisted of a rectangular acrylic chamber (17.5 × 6.5 × 6.5 cm) with metal grid flooring, divided into two 6.5 cm odor zones at the ends and a 4.5 cm neutral zone in the center. Odor zones were odorized by placing acrylic trays beneath the grid flooring with 500 µl of either the test odor or a control odor on a filter paper. Test and control odors were randomized between the two odor zones. For the test, a pup was placed in the center of the arena (i.e., neutral zone) and videotaped from above for 3 min using a camera (UI 2250-SE-M, IDS GMBH). Between each test, the chamber was cleaned with ethanol and allowed to dry. Odor–place preference tests were performed with pure odors (1% in mineral oil) or odor mixtures (90/10, 80/20 test/control odor). Mice were tracked using DeepLabCut (Mathis et al., 2018). The time spent over the different zones was quantified and analyzed in MATLAB.

To assess long-term neonatal odor memory, the dam was removed for 2 h on the following day (P11) for some of the mice tested on the day before, and the odor–place preference test was repeated with pure test versus control odor (1% in mineral oil).

Experimenters were blind for treatment group in behavioral tests. To reduce experimenter bias, analyses were performed using automated analysis methods (DeepLabCut for animal tracking, Deep Squeak for USV detection). The manual parts of the analyses were performed before group results were compared.

### Statistics

Statistical analysis was performed in MATLAB R2021a environment. Data were tested for normal distribution. Paired and unpaired *t* tests were used for normally distributed data, whereas nonparametric Wilcoxon rank sum and sign rank tests were used for non-normally distributed data to test for significant differences. Experiments with two factors were tested for significant differences using two-way ANOVA, with post hoc pairwise tests. Multiple comparisons were corrected with the Bonferroni method. Data are presented as violin plots or as mean ± standard error of the mean (SEM). Significance levels of *p < 0.05, **p < 0.01, or ***p < 0.001 were considered.

## Results

### High developmental DISC1 expression in OB projection neurons

The DISC1 protein is involved in the regulation of several developmental processes, such as progenitor proliferation, neuronal migration, and synapse formation (Kamiya et al., 2005; Mao et al., 2009; Ishizuka et al., 2011). The regulation of the formation and maintenance of synaptic connections by DISC1 is considered particularly important in the context of its association with neuropsychiatric disorders (Hayashi-Takagi et al., 2010; Brandon and Sawa, 2011). In adult mice, DISC1 is expressed in several brain areas including the OB, the hippocampus, and the cerebral cortex (Ma et al., 2002; Schurov et al., 2004 ; Meyer and Morris, 2008).

**Figure 1.**
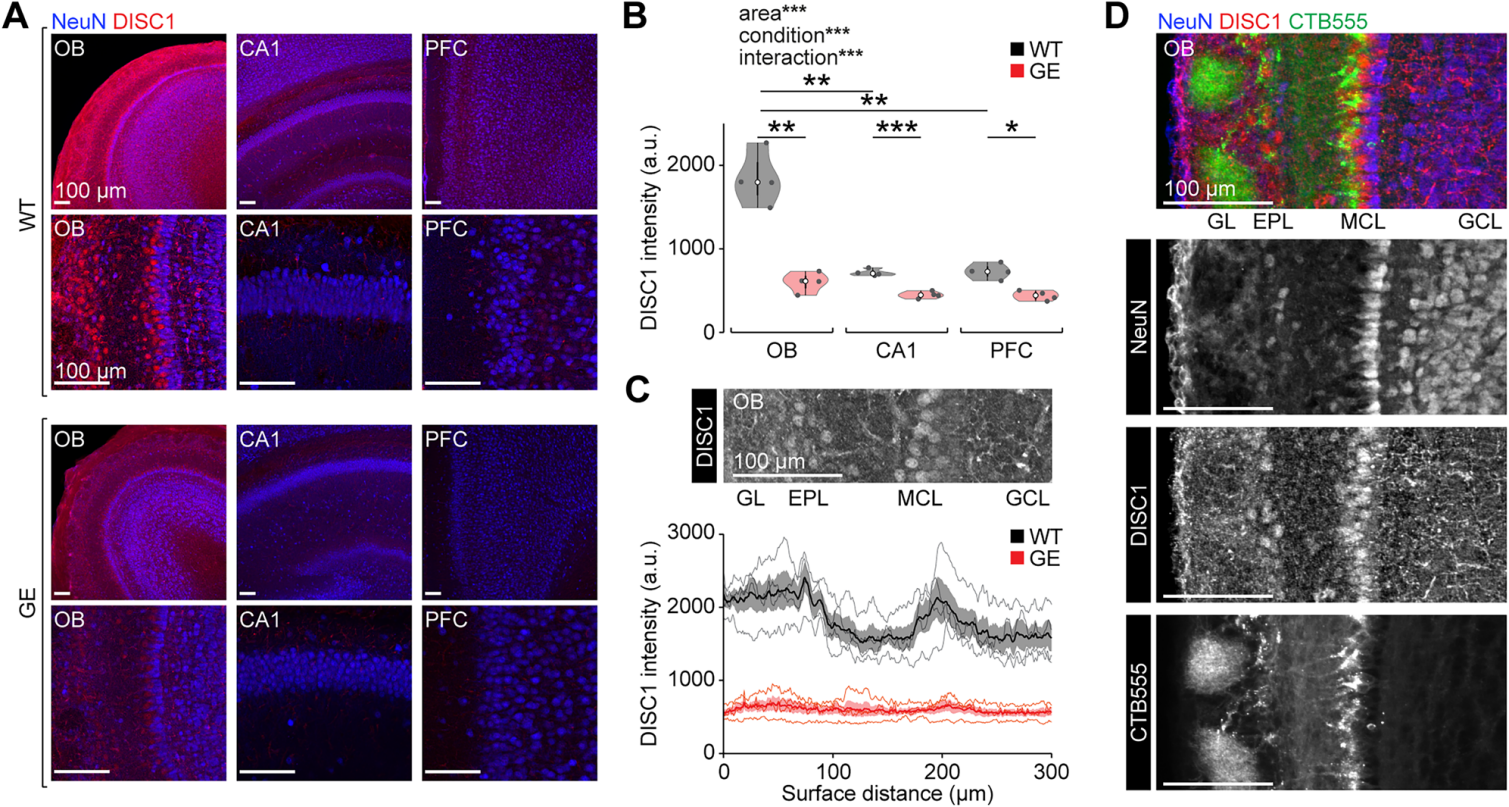
Strong expression of DISC1 in OB projection neurons at P10. ***A***, Coronal sections of OB, CA1, and PFC from P10 WT (top) and GE (bottom) mice immunostained for NeuN (blue) and DISC1 (red) at low and high magnification. Slices from different areas were stained in parallel and images were acquired with identical settings. ***B***, Fluorescence intensity of DISC1 immunolabeling in OB, CA1, and PFC in P9–10 WT (*n* = 4) and GE (*n* = 4) mice. Two-way ANOVA revealed significant effects of animal condition (*F*_(1)_ = 97.7; *p* = 1.07 × 10^−8^), area (*F*_(2)_ = 49.2; *p* = 5.03 × 10^−8^), and their interaction (*F*_(2)_ = 28.0; *p* = 2.97 × 10^−6^). ***C***, Top, Coronal section of OB from a P10 WT mouse immunostained for DISC1. Bottom, spatially resolved DISC1 intensity in P9–10 WT (*n* = 4) and GE (*n* = 4) mice. ***D***, Coronal section of OB from a P10 WT mouse immunostained for DISC1 with OB projection neurons labeled by injection of the retrograde tracer CTB555 into the piriform cortex. Shaded areas in C correspond to SEM. Significant differences are indicated as *, **, and *** for *p* < 0.05, 0.01, and 0.001, respectively. GL glomerular layer; EPL external plexiform layer; MCL mitral cell layer; GCL granule cell layer.

As a first test for a potential role of the olfactory system in the pathophysiology of GE mice, we investigated DISC1 expression during postnatal development. Using immunohistochemistry, we found high expression levels of DISC1 in the OB of WT mice at the beginning of the second postnatal week (Fig. 1*A*,*B*). DISC1 expression was significantly higher in the OB compared with the hippocampal subdivision CA1 (*p* = 3.96 × 10^−3^) and the PFC (*p* = 4.97 × 10^−3^) in WT mice. In GE mice, DISC1 expression was significantly reduced compared with WT controls in all areas (OB *p* = 3.15 × 10^−3^; CA1 *p* = 8.44 × 10^−4^; PFC *p* = 0.0157). More detailed examination of the OB revealed increased DISC1 expression in the neuronal processes but also in the cell bodies of neurons in the MCL and the EPL (Fig. 1*C*). This is indicative for a strong DISC1 expression in mitral/tufted cells, the projection neurons of the OB. Immunostaining of OB slices of P10 WT mice, injected with the retrograde tracer CTB555 into the piriform cortex at P5, confirmed the expression of DISC1 in OB projection neurons (Fig. 1*D*).

Together, these results support the idea of a potential developmental dysfunction of the OB in GE mice.

### Reduced spontaneous OB activity in immune-challenged *Disc1^+/−^* mice during development

**Figure 2.**
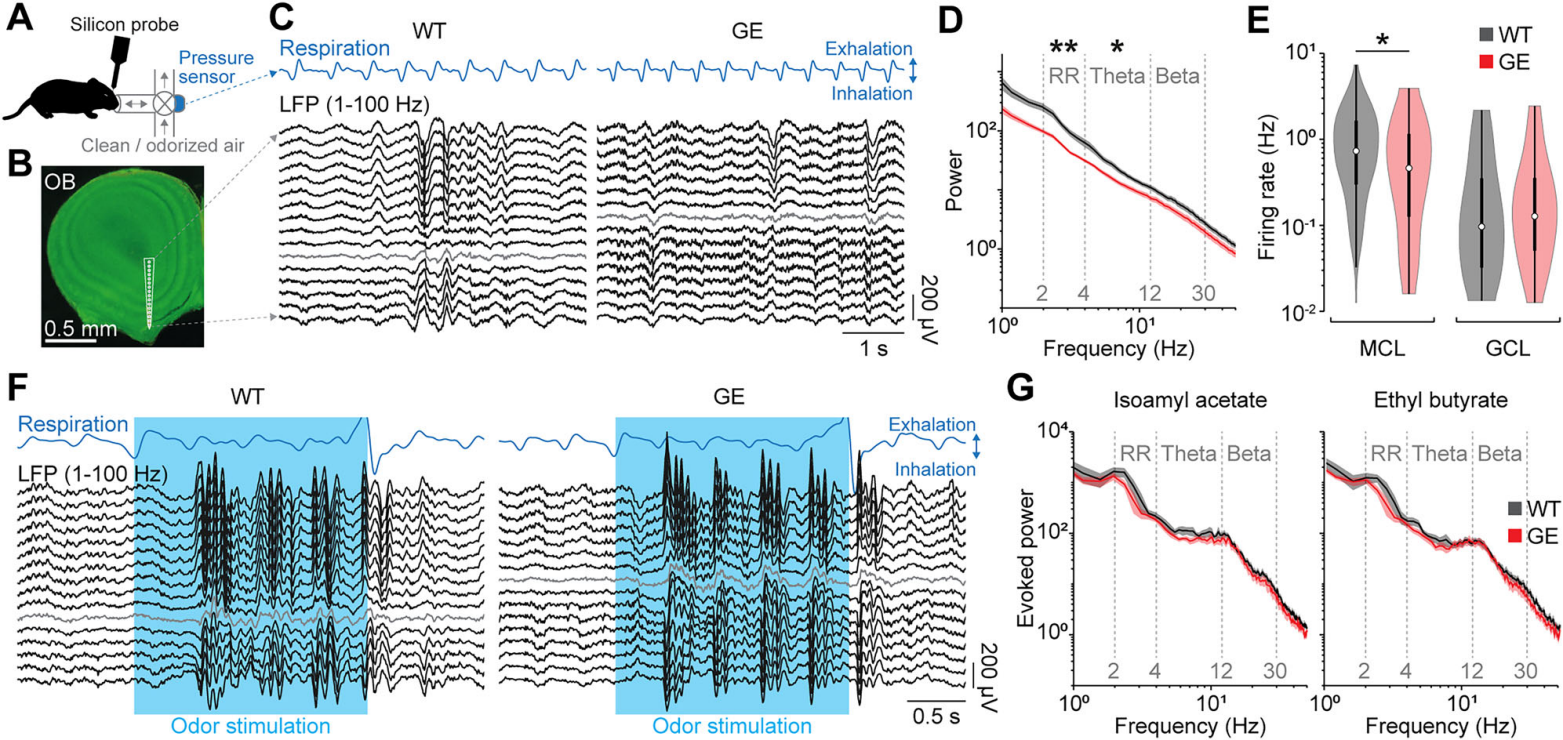
Reduced spontaneous, but normal odor-evoked activity in the OB of immune-challenged *Disc1^+/−^* mice at P8–10. ***A***, Experimental setup for recordings of spontaneous and respiration-triggered odor–evoked activity in the OB of P8–10 mice. ***B***, Example coronal section with a reconstruction of the DiI-labeled silicon probe tip in the ventral OB. ***C***, Example extracellular recording of spontaneous activity from the ventral OB of a P10 WT and GE mouse using a silicon probe with 16 recording sites spanning across the MCL (gray). Down- and upward deflections on the respiration trace from the pressure sensor indicate inhalation and exhalation, respectively. ***D***, Power spectra of spontaneous OB activity in P8–10 WT (*n* = 17) and GE (*n* = 14) mice. ***E***, The firing rate of spontaneous OB activity in P8–10 WT and GE mice for units recorded in MCL (WT *n* = 172; GE *n* = 49 units) and GCL (WT *n* = 55; GE *n* = 38 units). ***F***, Same as ***C*** for odor-evoked activity. ***G***, Power spectra of odor-evoked OB activity in P8–10 WT (*n* = 11) and GE (*n* = 11) mice. Shaded areas in ***D*** and ***G*** correspond to SEM. Significant differences are indicated as *, **, and *** for *p* < 0.05, 0.01, and 0.001, respectively.

To investigate developmental OB activity in GE mice, we performed in vivo electrophysiological recordings using multisite silicon probes. We monitored spontaneous and odor-evoked activity in the ventral OB of WT and GE mice at P8–10 (Fig. 2*A*,*B*). The extracellular recordings were combined with respiration measurements using a pressure sensor. Both WT and GE mice showed continuous activity in the LFP recorded in the OB with the typical dominant RR (2–4 Hz) that reverses polarity at the MCL (Fig. 2*C*). However, the power in RR and theta (4–12 Hz) frequency bands were significantly reduced in GE mice (RR *p* = 1.53 × 10^−3^; theta *p* = 0.012), whereas the power in beta (12–30 Hz) was not significantly different (*p* = 0.27; Fig. 2*D*). Furthermore, the firing rate of single units in the OB was reduced in GE mice, particularly for neurons in the MCL (MCL *p* = 0.046; GCL *p* = 1.0), indicating a reduction in the activity of OB projection neurons in the absence of odor stimulation (Fig. 2*E*).

Next, we used the respiration measurement for closed-loop respiration–triggered odor stimulation to investigate odor-evoked activity in the OB of WT and GE mice. Odor stimulation with the pure odorants isoamyl acetate or ethyl butyrate (1% v/v in mineral oil) was triggered by exhalations to guarantee for stable odor presentation at the subsequent inhalation. Odor presentation induced strong activation of the OB in WT and GE mice (Fig. 2*F*). In contrast to spontaneous activity, odor-evoked activity was similar for WT and GE mice across all frequency bands (isoamyl acetate, RR *p* = 0.264; theta *p* = 1.0; beta *p* = 0.96; ethyl butyrate, RR *p* = 0.36; theta *p* = 0.87; beta *p* = 1.0; Fig. 2*G*).

**Figure 3.**
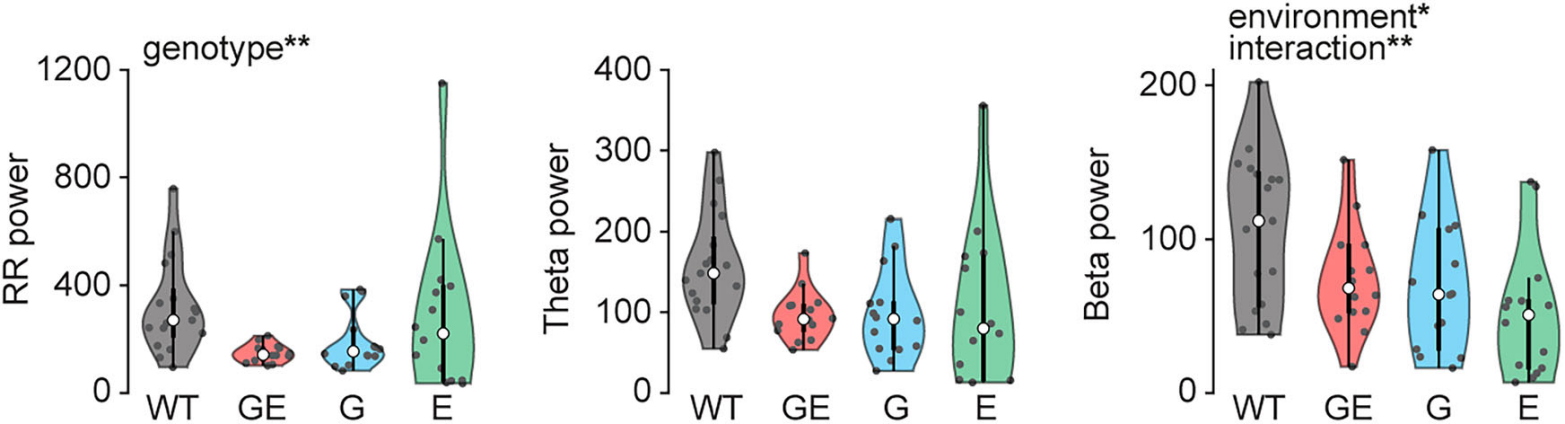
Distinct patterns of spontaneous OB activity in *Disc1^+/−^* and in immune-challenged mice at P8–10. Power of spontaneous OB activity quantified in different frequency bands (left, RR; middle, theta; right, beta) in P8–10 WT (*n* = 17), GE (*n* = 14), G (*n* = 14), and E (*n* = 14) mice. For RR power, two-way ANOVA revealed a significant effect of genotype (*F*_(1)_ = 8.05; *p* = 6.4 × 10^−3^). For theta power, two-way ANOVA revealed no significant effects, with the effect of genotype being close to significance (*F*_(1)_ = 3.71; *p* = 0.059). For beta power, two-way ANOVA revealed significant effects of environment (*F*_(1)_ = 5.01; *p* = 0.029) and the interaction of genotype and environment (*F*_(1)_ = 7.6; *p* = 7.9 × 10^−3^). Significant differences are indicated as *, **, and *** for *p* < 0.05, 0.01, and 0.001, respectively.

Notably, mice that only carry the genetic mutation (G) or the environmental hit (E) had distinct effect on OB activity. We found a significant effect of the genetic mutation on power in RR, whereas power in beta frequency was affected by the environmental factor and the interaction of gene and environment (Fig. 3).

Thus, odor stimulation evokes activity in the OB of GE mice that is similar to WT controls, whereas spontaneous activity in the absence of odor stimulation is significantly reduced in GE mice.

### Reduced OB activity results in weaker drive of the hippocampal–prefrontal network

**Figure 4.**
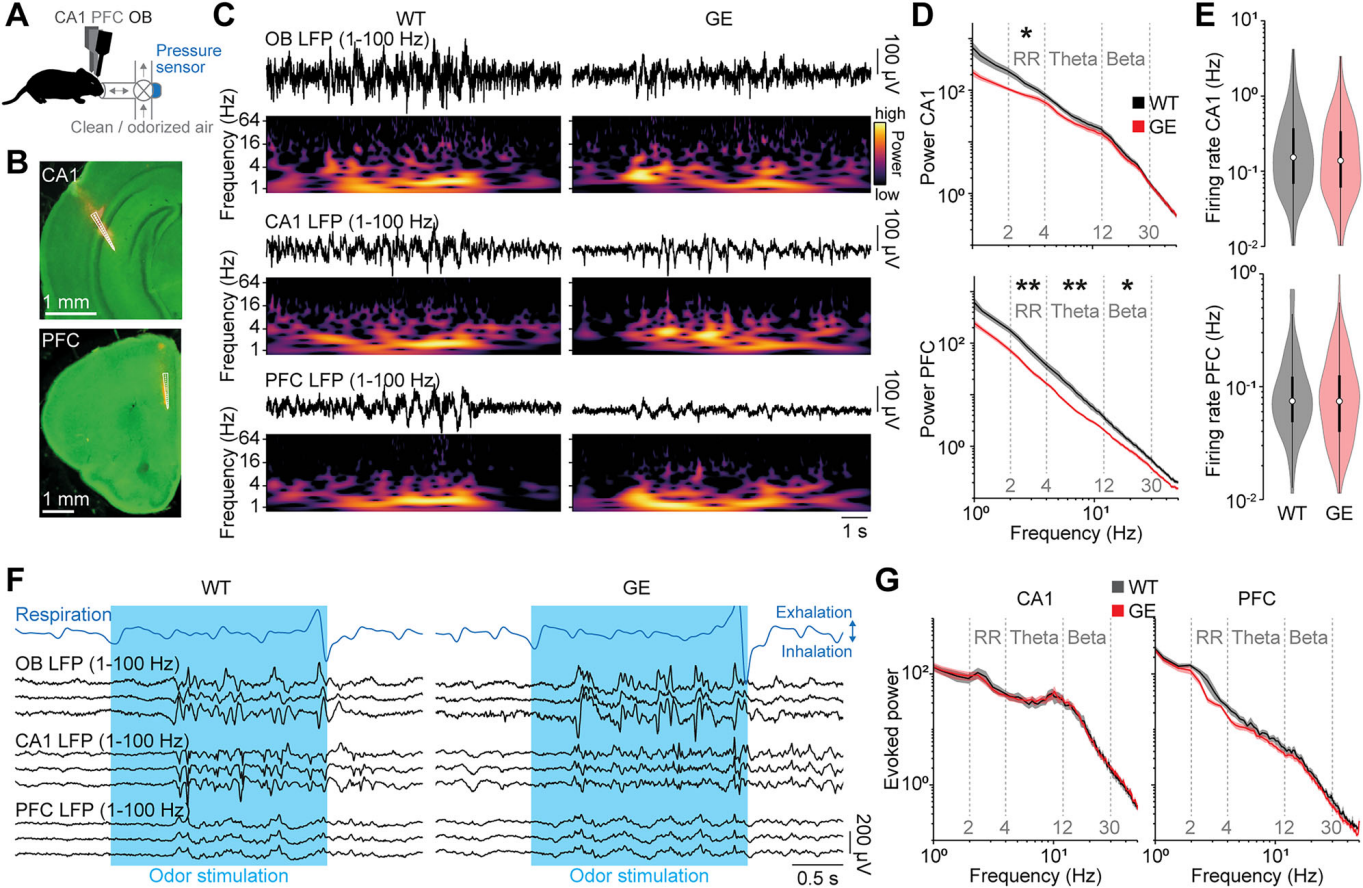
Reduced activity in the hippocampal–prefrontal network in immune-challenged *Disc1^+/−^* mice at P8–10. ***A***, Experimental setup for triple recordings of spontaneous and respiration-triggered odor–evoked activity in OB, CA1, and PFC of P8–10 mice. ***B***, Example coronal sections with a reconstruction of the DiI-labeled silicon probe tips in CA1 of the intermediate hippocampus (top) and the medial part of the PFC (bottom). ***C***, Examples of spontaneous LFP activity recorded simultaneously from OB, CA1, and PFC of a P10 WT and GE mouse and the corresponding wavelet spectra. ***D***, Power spectra of spontaneous CA1 and PFC activity in P8–10 WT (CA1 *n* = 15; PFC *n* = 14) and GE (CA1 *n* = 14; PFC *n* = 14) mice. ***E***, The firing rate of spontaneous CA1 and PFC single unit activity in P8–10 WT (CA1 *n* = 270; PFC *n* = 167 units) and GE (CA1 *n* = 182; PFC *n* = 153 units) mice. ***F***, Examples of odor-evoked LFP activity recorded simultaneously from OB, CA1, and PFC of a P10 WT and GE mouse. ***G***, Power spectra of odor-evoked CA1 and PFC activity in P8–10 WT (*n* = 11) and GE (*n* = 11) mice. Shaded areas in ***D*** and ***G*** correspond to SEM. Significant differences are indicated as *, **, and *** for *p* < 0.05, 0.01, and 0.001, respectively.

Does altered activity in the OB of GE mice affect activity in downstream areas? To address this question, we performed simultaneous recordings from OB, CA1 of the intermediate hippocampus, and the medial PFC of P8–10 WT and GE mice (Fig. 4*A*,*B*). While OB activity was already continuous at this age, CA1 and PFC showed discontinuous patterns of electrical activity (Fig. 4*C*) characteristic for this age (Brockmann et al., 2011). As reported in our previous publications (Hartung et al., 2016; Chini et al., 2020), prefrontal LFP power in theta and beta frequency range was reduced (theta *p* = 2.6 × 10^−3^; beta *p* = 0.028) in GE mice when compared with WT controls, whereas no differences were detected for CA1 (theta *p* = 0.57; beta *p* = 1.0; Fig. 4*D*). Of note, the power in RR frequency band was reduced in both areas (CA1 *p* = 0.045; PFC *p* = 4.8 × 10^−3^) for GE mice in line with reduced RR power in the OB. Firing rates of single units in CA1 (*p* = 0.41) and PFC (*p* = 0.65) were similar for WT and GE (Fig. 4*E*). In this study, mostly deep layers of the PFC were recorded, which explains the lack of altered firing rates that are characteristic for prefrontal layer 2/3 in neonatal GE mice (Chini et al., 2020).

Next, we tested whether there is a difference in the propagation of odor-evoked activity to the hippocampal–prefrontal network in GE mice. Odor stimulation evoked pronounced activation in CA1 and PFC of WT and GE mice (Fig. 4*F*). As for OB, we found no significant differences for odor-evoked activity in CA1 and PFC of P8–10 WT and GE mice (Fig. 4*G*).

Thus, similar to the OB, spontaneous activity in CA1 and PFC of P8–10 GE mice is decreased, yet odor-induced activity is similar to age-matched WT controls.

**Figure 5.**
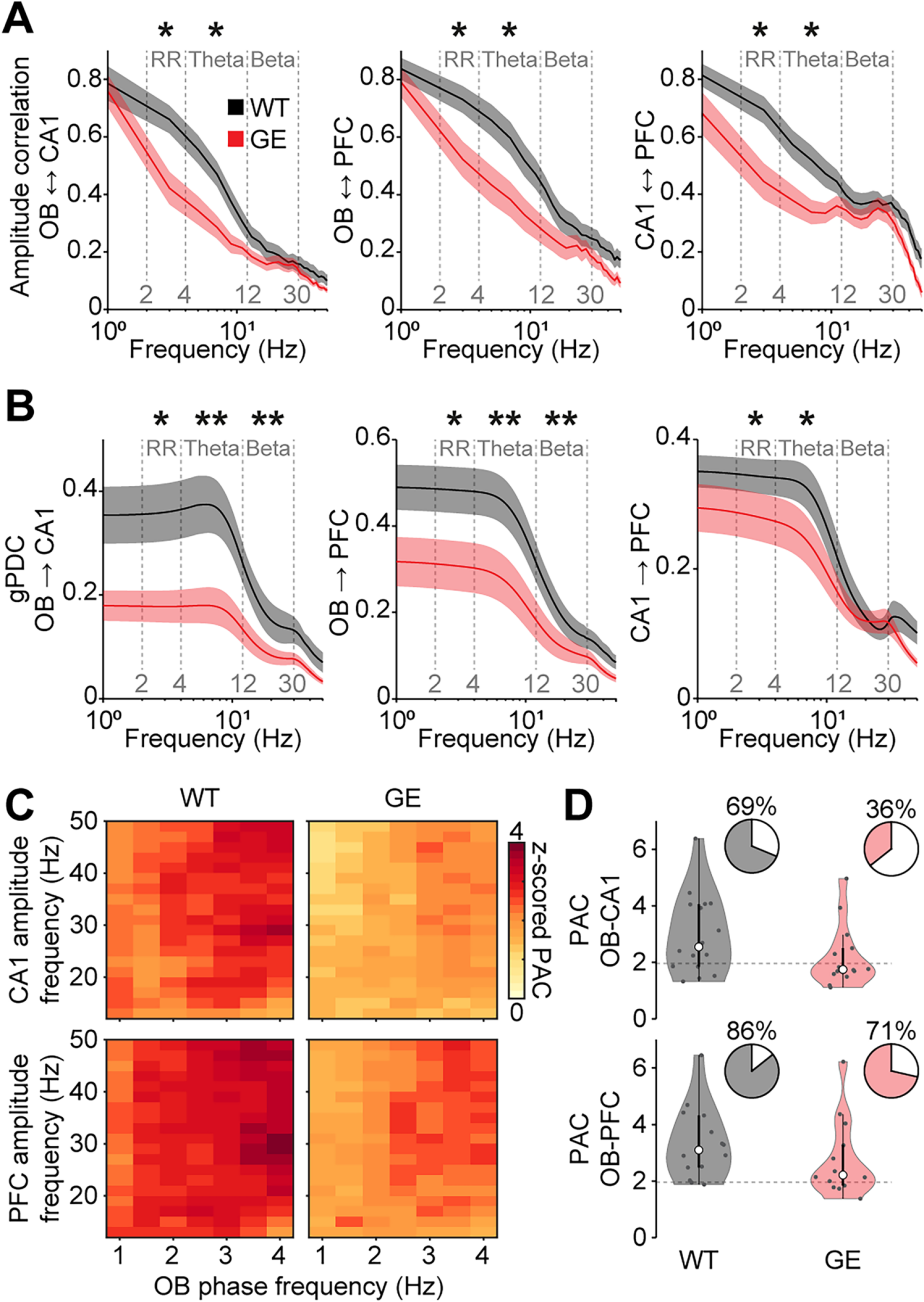
Reduced drive of the hippocampal–prefrontal network in immune-challenged *Disc1^+/−^* mice at P8–10. ***A***, Frequency-resolved amplitude correlation between OB-CA1 (WT *n* = 16; GE *n* = 14), OB-PFC (WT *n* = 14; GE *n* = 14), and CA1-PFC (WT *n* = 13; GE *n* = 14) for P8–10 WT and GE mice. ***B***, Frequency-resolved gPDC from OB to CA1 (WT *n* = 16; GE *n* = 14), from OB to PFC (WT *n* = 14; GE *n* = 14), and from CA1 to PFC (WT *n* = 13; GE *n* = 14) for P8–10 WT and GE mice. ***C***, Color-coded average PAC of CA1 (WT *n* = 16; GE *n* = 14) and PFC (WT *n* = 14; GE *n* = 14) LFP amplitude at fast frequencies (12–50 Hz) to slow-frequency oscillations (1–4 Hz) in OB for P8–10 WT and GE mice. ***D***, *Z*-scored PAC of CA1 and PFC LFP amplitude at fast frequencies to slow frequencies in the OB for P8–10 WT and GE mice. Pie charts show the percentage of recordings with significant coupling. Dotted lines correspond to a *z*-score of 1.96 indicating the significance level. Shaded areas in ***A*** and ***B*** correspond to SEM. Significant differences are indicated as *, **, and *** for *p* < 0.05, 0.01, and 0.001, respectively.

Reduced RR power in OB, CA1, and PFC of GE mice during development suggests a possible reduction in the drive from the OB to the hippocampal–prefrontal network. To test this hypothesis, we used pairwise analyses of the simultaneously recorded areas to test their functional interactions in P8–10 WT and GE mice. First, we calculated the amplitude correlation of the LFP between areas as a measure for nondirected interactions that is based on the similarity of the amplitude fluctuations at a given frequency. We found a significant reduction in the interaction between OB and CA1 (RR *p* = 0.018; theta *p* = 0.012; beta *p* = 0.49), OB and PFC (RR *p* = 0.045; theta *p* = 0.023; beta *p* = 0.063), as well as CA1 and PFC (RR *p* = 0.011; theta *p* = 0.039; beta *p* = 0.37) in RR and theta frequency but not in beta frequency for GE mice (Fig. 5*A*). Next, we tested more directed measures of functional interactions. gPDC, which assesses the directionality of pairwise interactions, revealed a strong reduction in the drive from OB to CA1 (RR *p* = 0.011; theta *p* = 0.0044; beta *p* = 0.0037) and OB to PFC (RR *p* = 0.011; theta *p* = 0.0051; beta *p* = 0.0016) in all frequency bands for GE mice (Fig. 5*B*). The directed interaction from CA1 to PFC was reduced in RR (*p* = 0.024) and theta (*p* = 0.018) but not beta (*p* = 0.76) frequency for GE mice, as previously reported (Xu et al., 2021b). Finally, we quantified PAC to evaluate the cross-frequency modulation of oscillatory power at fast frequencies (12–50 Hz) in CA1 and PFC by the slow RR generated in the OB. The strength of PAC from OB to CA1 and PFC was reduced in GE mice, as was the percentage of recordings with significant cross-frequency coupling (OB to CA1, WT 11 of 16, GE 5 of 14 mice; OB to PFC, WT 12 of 14, GE 10 of 14 mice; Fig. 5*C*,*D*).

Together, these findings show that the drive from OB to CA1 and PFC is reduced in GE mice.

### Inhibiting OB output or OB-confined DISC1 knockdown reduces hippocampal–prefrontal activity

**Figure 6.**
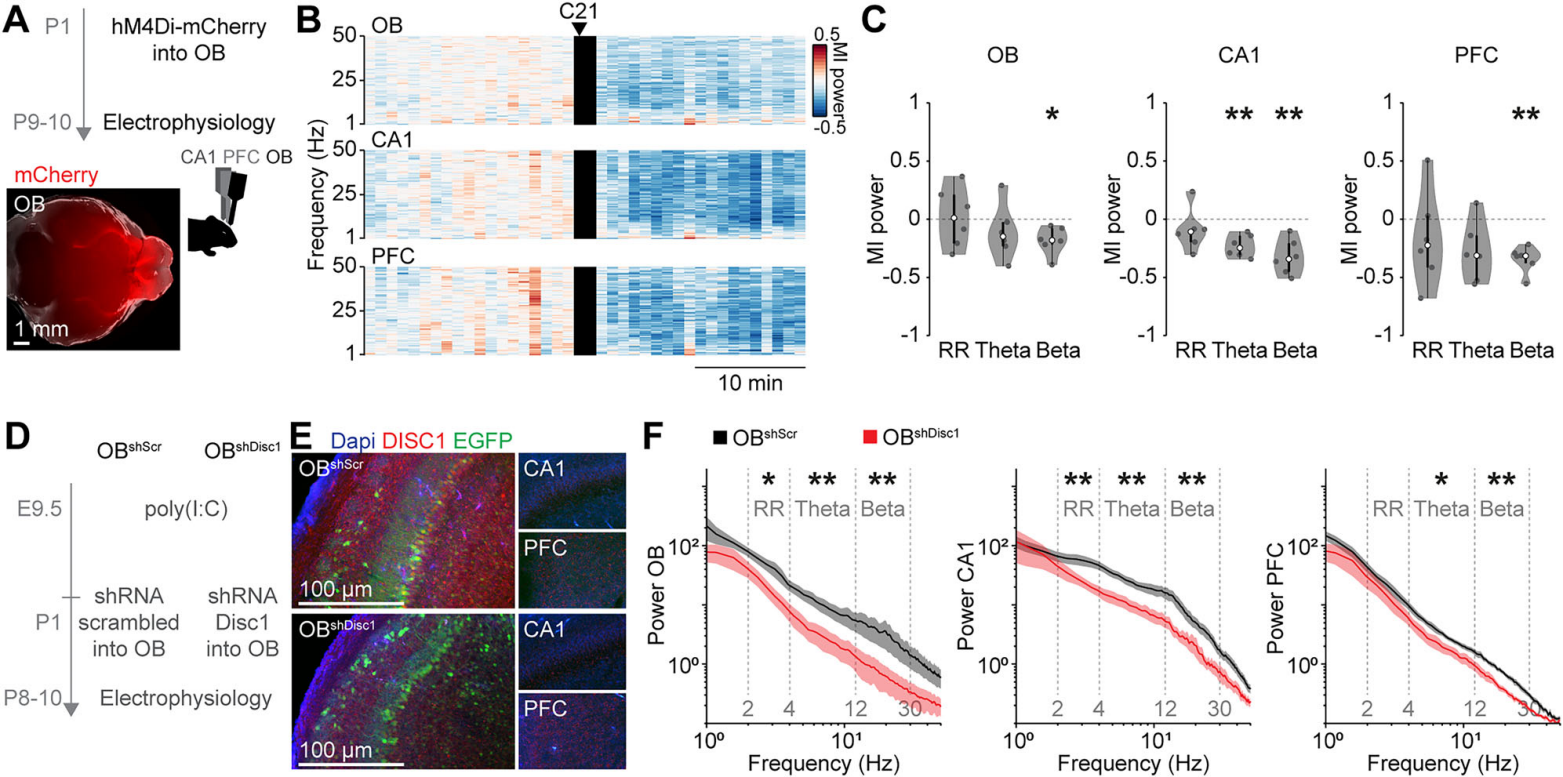
Chemogenetic or DISC1 knockdown-mediated reduction of OB activity decreases hippocampal–prefrontal network activity. ***A***, Top, Experimental timeline for hM4Di-mediated inhibition of OB outputs during electrophysiological recordings in P9–10 Tbet-cre mice. Bottom, A representative image of the ventral brain of a P10 mouse showing hM4Di-mCherry expression labeling the OB and mitral/tufted cell axons in the lateral olfactory tract. ***B***, Color-coded averaged MI of LFP power spectra for OB, CA1, and PFC for P9–10 mice (*n* = 6) relative to C21 injection activating the inhibitory DREADD hM4Di in OB mitral/tufted cells. ***C***, MI power in distinct frequency bands before (pre) and after (post) injection of P21 for OB, CA1, and PFC. ***D***, Experimental timeline for shRNA-mediated knockdown of DISC1 in the OB of immune-challenged mice. ***E***, Coronal sections of the OB, CA1, and PFC of immune-challenged mice at P9 after P1 injection of AAVs encoding shRNA against Disc1 (OB^shDisc1^) or a scrambled control (OB^shScr^) immunostained for DISC1 (red). EGFP (green) expression is mediated by the AAVs. ***F***, Power spectra of spontaneous OB, CA1, and PFC activity in P8–10 OB^shDisc1^ (*n* = 8) and OB^shScr^ (*n* = 8) immune-challenged mice. Shaded areas in ***F*** correspond to SEM. Significant differences are indicated as *, **, and *** for *p* < 0.05, 0.01, and 0.001, respectively.

Next, we used two experimental approaches to directly test the role of OB activity and DISC1 expression in the OB for the hippocampal–prefrontal network. First, we used chemogenetic manipulations to acutely inhibit the OB output in neonatal mice and investigated the consequences on the network activity in CA1 and PFC. AAV9-EF1a-DIO-hM4Di-mCherry was injected bilaterally into the OB of Tbet-cre mice at P1, resulting in expression of the inhibitory DREADD hM4Di in mitral/tufted cells, the projection neurons of the OB (Fig. 6*A*). Activation of hM4Di by injection of C21 to inhibit OB outputs was performed simultaneously with extracellular recordings from OB, CA1, and PFC at P9–10. The changes in oscillatory power after C21 injections were quantified for each frequency band. For this, we calculated the modulation index (MI) as the power difference after and before injection divided by the sum of the two. We found that inhibiting OB output reduced the activity in OB (RR *p* = 1.0; theta *p* = 0.89; beta *p* = 0.040), as well as in CA1 (RR *p* = 0.79; theta *p* = 0.0058; beta *p* = 0.0099) and PFC (RR *p* = 1.0; theta *p* = 0.13; beta *p* = 0.0022; Fig. 6*B*,*C*). These results indicate that OB activity is an important driver for the activation of hippocampal–prefrontal circuits.

Second, we used shRNA-mediated knockdown to assess the specific role of OB-expressed DISC1 for the impaired hippocampal–prefrontal activity in GE mice. For this, AAV9 encoding for EGFP and shRNA against *Disc1* (OB^shDisc1^) or a scrambled control (OB^shScr^) were injected bilaterally into the OB of immune-challenged WT mice at P1 (Fig. 6*D*). This resulted in an OB-specific knockdown of DISC1 for OB^shDisc1^ during early development, but did not change DISC1 expression in CA1 and PFC (Fig. 6*E*). OB-confined DISC1 knockdown resulted in a broadband reduction of LFP power in the OB (RR *p* = 0.015; theta *p* = 0.0047; beta *p* = 0.0029), CA1 (RR *p* = 0.0047; theta *p* = 0.0029; beta *p* = 0.0070), and PFC (RR *p* = 0.083; theta *p* = 0.028; beta *p* = 0.0011) of P8–10 mice (Fig. 6*F*).

Thus, OB-confined DISC1 knockdown during early development is sufficient to reduce not only OB activity but also network activity in the hippocampal–prefrontal circuit in immune-challenged mice.

### Normal odor detection but impaired odor memory in immune-challenged Disc1^+/−^ mice

**Figure 7.**
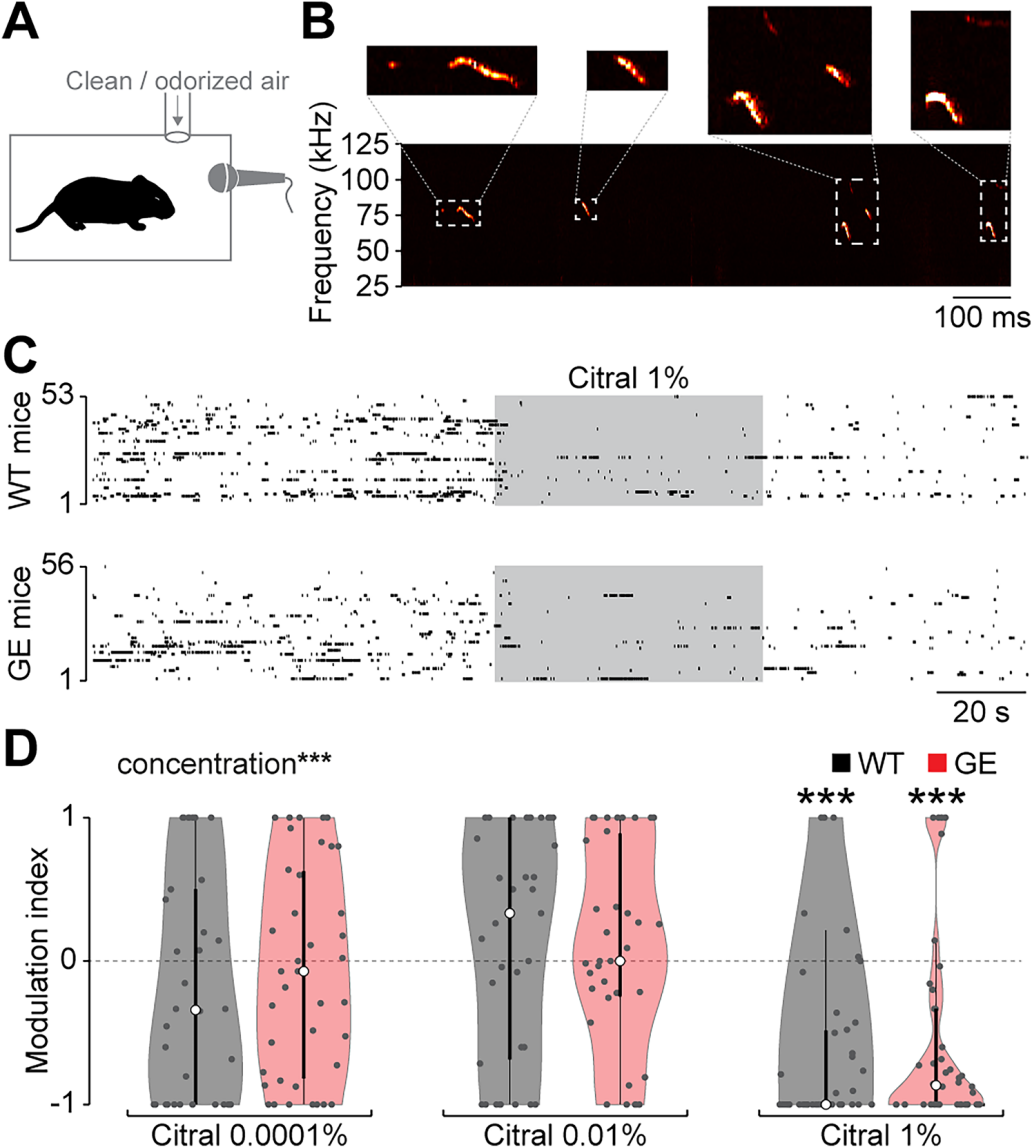
Normal odor detection in immune-challenged *Disc1^+/−^* mice at P9. ***A***, Experimental setup for USV recordings during odor exposure. ***B***, Example spectrogram of USVs of a P9 mouse. ***C***, The raster plot of USV suppression in response to the odorant citral for P9 WT (*n* = 53) and GE (*n* = 56) mice. Each line represents one mouse. ***D***, MI of USV numbers defined as the (call rate during odor − before odor) / (during odor + before odor) in response to the odorant citral at different concentrations. Two-way ANOVA revealed significant effects of concentration (*F*_(2)_ = 21.37; *p* = 2.4 × 10^−9^), but not of animal condition or their interaction. Significant differences are indicated as *, **, and *** for *p* < 0.05, 0.01, and 0.001, respectively.

Normal propagation of odor-evoked activity from the OB to the hippocampal–prefrontal network suggests that GE mice might have normal odor processing during development. To address this hypothesis, we recorded USVs of P9 WT and GE mice when exposed to the odorant citral. Citral triggers an innate aversive response and reduces USV calls in neonatal mice, similar to the odor of adult males (Lemasson et al., 2005). Pups were placed in a small chamber with a continuous flow of clean air for a baseline period after which citral was added to the airstream (Fig. 7*A*–*C*). This procedure was repeated with increasing concentrations of citral. While concentrations of 0.0001 and 0.01% of citral (v/v in mineral oil) did not reduce call rates, both WT and GE mice similarly reduced their call rate in response to citral at 1% (WT *p* = 6.6 × 10^−4^; GE *p* = 2.0 × 10^−5^; Fig. 7*D*).

Thus, no evidence for impaired simple odor detection was found in developing GE mice, consistent with normal odor-evoked activity.

**Figure 8.**
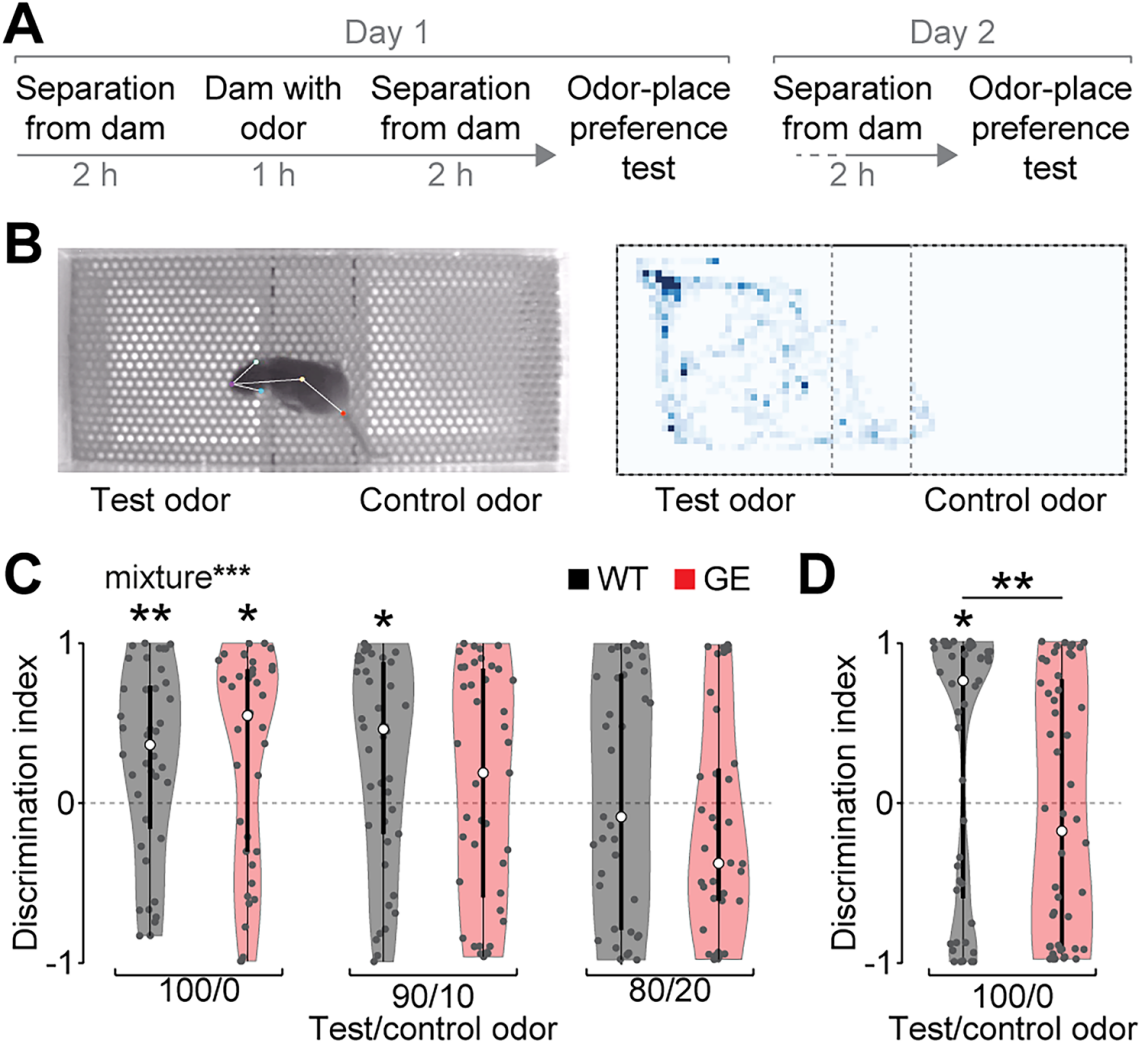
Impaired long-term odor memory in immune-challenged *Disc1^+/−^* mice at P10/11. ***A***, Timeline of neonatal odor learning and odor–place preference test for P10–11 mice. ***B***, Left, An example image of a P10 mouse in the odor–place preference test chamber. Right, Example color-coded position of a mouse's nose traced by DeepLabCut during the odor–place preference test. ***C***, Discrimination index of test and control odor defined as (time in test zone + time in control zone) / (time in test zone + time in control zone) in the odor–place preference test on the same day of neonatal odor learning for P10 WT and GE mice. Test and control odors were presented pure (100/0, WT *n* = 40; GE *n* = 38 mice) or mixed (90/10, WT *n* = 42; GE *n* = 41 mice; 80/20, WT *n* = 37; GE *n* = 36 mice). Two-way ANOVA revealed significant effects of odor mixture (*F*_(2)_ = 4.54; *p* = 0.011), but not of animal condition or their interaction. ***D***, Discrimination index of test and control odor in the odor–place preference test the day after neonatal odor learning for P11 WT (*n* = 57) and GE (*n* = 52) mice. Significant differences are indicated as *, **, and *** for *p* < 0.05, 0.01, and 0.001, respectively.

We recently reported that transient inhibition of OB outputs from P8 to P10 in WT mice perturbs the functional maturation of the hippocampal formation and results in long-lasting cognitive deficits (Chen et al., 2023). Thus, we hypothesized that the reduced spontaneous OB activity and drive to the hippocampal–prefrontal network in developing GE mice might cause similar impairments. We used neonatal odor learning, a one-trial associative odor learning task for mouse pups (Armstrong et al., 2006), to test the learning and memory abilities in P10–11 WT and GE mice (Fig. 8*A*). For this test, the dam was separated from the pups for 2 h. Subsequently, a novel odor was applied to the teats before the dam was returned to the pups. The separation period guarantees feeding of the pups soon after odor application such that an association between food consumption and the odor can be formed. After a second separation period, pups were tested in an odor–place preference test with the learned test odor and a novel control odor (Fig. 8*B*). Isoamyl acetate and ethyl butyrate (1% in mineral oil) were randomly assigned as test and control odor for each litter, and the position of the test odor was randomized for each pup.

Both WT and GE mice spend more time on the side of the test odor when pure odors were presented in the odor–place preference test (WT *p* = 7.5 × 10^−3^; GE *p* = 0.021), and no difference was found between the groups (Fig. 8*C*). To increase the difficulty of the test, we presented mice with a mixture of test and control odors during the odor–place preference test. Interestingly, only WT controls were able to distinguish the learned test odor at a mixture of 90/10% (WT *p* = 0.015; GE *p* = 0.31), but there was no significant group difference for WT and GE mice. Neither WT nor GE mice showed a place preference when odors were presented at a mixture of 80/20% (WT *p* = 0.48; GE *p* = 0.22). These results indicate that odor learning is not impaired in GE mice at P10. However, when the odor–place preference test was done with a 24 h delay period after neonatal odor learning, WT pups strongly preferred the learned test odor (*p* = 0.012), whereas GE mice did not distinguish between test and control odor (*p* = 0.18), and their performance was significantly reduced compared with WT mice (*p* = 2.6 × 10^−3^; Fig. 8*D*).

Together, these data revealed that odor detection and learning appear largely normal in developing GE mice, whereas their long-term memory is impaired.

## Discussion

In this study, we examined early OB activity and its influence on developing hippocampal–prefrontal networks in GE mice. We found strong DISC1 expression in OB projection neurons during development, which was significantly reduced in GE mice. GE pups displayed reduced spontaneous activity in the OB, whereas odor-evoked activity was comparable to WT controls. Correspondingly, the drive from the OB to the hippocampal–prefrontal network was reduced for spontaneous activity, but the propagation of odor-evoked activity to hippocampal CA1 and PFC was not altered. Consistent with these findings, we found no evidence for altered odor detection and learning in GE mouse pups, but their long-term odor memory was impaired. Furthermore, acute inhibition of OB outputs reduced the oscillatory activity in hippocampal–prefrontal networks and an OB-confined knockdown of DISC1 during early postnatal development impairs activity in CA1 and PFC. We conclude that reduced spontaneous activity in the OB might contribute to the altered maturation of the hippocampal–prefrontal network as well as memory deficits previously described in GE mice (Hartung et al., 2016; Chini et al., 2020).

During development, spontaneous and sensory-evoked activity is required for the refinement of immature neuronal networks. Neuronal activity influences a range of developmental processes such as neuronal survival and dendritic growth, as well as synapse formation and pruning (Katz and Shatz, 1996; Kirkby et al., 2013; Kirischuk et al., 2017; Bitzenhofer et al., 2021). The spontaneous and odor-induced OB activity is a major drive for developing hippocampal–prefrontal networks (Gretenkord et al., 2019; Kostka et al., 2020; Kostka and Hanganu-Opatz, 2023). Transient inhibition of OB outputs at the beginning of the second postnatal week disrupts the functional maturation of the hippocampal formation, which causes long-lasting deficits in cognitive abilities (Chen et al., 2023). Here, we show that the reduction of spontaneous activity in the OB of GE mice appears to have similar consequences for the maturation of the hippocampal–prefrontal network. We found a significant reduction in the activity of OB projection neurons and a concurrent decrease in the coordinated activity in slow oscillatory rhythms in GE mice. Particularly, the power in RR, oscillatory activity within 2–4 Hz, was significantly reduced in OB, as well as in CA1 and PFC. This rhythm is driven by repetitive input to the OB generated by the inhalation–exhalation cycle and coordinates activity in downstream areas in neonatal and adult mice (Biskamp et al., 2017; Gretenkord et al., 2019; Karalis and Sirota, 2022; Basha et al., 2023; Kostka and Hanganu-Opatz, 2023). Reduced coordination of activity in CA1 and PFC as a result of a reduced coordination of OB activity by respiration in GE mice might underlie the detrimental effect on the maturation of the hippocampal–prefrontal network. Interestingly, the single-hit mouse model carrying the genetic disruption of *Disc1^+/−^* without the environmental hit showed a similar reduction of RR power in OB, indicating that the genetic deficit alone is sufficient for RR impairment in the primary olfactory area, whereas the environmental hit and the GE interaction seem to cause reduced activity in beta frequency. In contrast to this, we previously reported that oscillatory activity in CA1 and PFC is only affected when the *Disc1^+/−^* mutation is combined with maternal immune activation (Oberlander et al., 2019). This difference might be linked to the strong expression of DISC1 in the developing OB of WT mice. We found that acute inhibition of OB outputs reduces network activity in CA1 and PFC in WT mice. The absence of an effect on network activity in CA1 and PFC in mice that only carry the genetic mutation (Oberlander et al., 2019) might be explained by differences in the strength and time course in the reduction of OB activity. We interpret the data such that the genetic hit is sufficient to reduce activity in the OB, but only the combination of the genetic and the environmental factors results in impaired activity in the hippocampal–prefrontal network.

DISC1 is also expressed in CA1 and PFC during development and previous studies found that specific knockdown of DISC1 in these areas in combination with maternal immune activation suffices to impair hippocampal–prefrontal activity (Xu et al., 2019, 2021a). However, strong DISC1 expression in OB projection neurons and high levels of OB activity during development in WT mice indicate a particular role of reduced OB activity for the disturbed maturation of the hippocampal–prefrontal network in GE mice. Transient inhibition of OB outputs during postnatal development disrupts the functional maturation of the hippocampal formation (Chen et al., 2023). Consistent with these findings, we found that an OB-confined knockdown of DISC1 results in reduced activity in the OB, CA1, and PFC in immune-challenged mice. Thus, we conclude that reduced functional DISC1 protein in the OB of GE mice contributes to the reduction of activity in the hippocampal–prefrontal network.

The clear directionality in the drive of activity from OB to CA1 and PFC is consistent with anatomical data showing that feedback projections to the OB develop late and are still sparse at the beginning of the second postnatal week (Kostka and Bitzenhofer, 2022b). The axons of OB projection neurons are bundled in the lateral olfactory tract that distributes olfactory information to a range of brain areas (Igarashi et al., 2012). While the OB has no direct projections to CA1 and PFC in mice, strong projections through the piriform cortex and the lateral entorhinal cortex provide a short pathway from the OB to the hippocampal–prefrontal network. This pathway is functional early during development (Gretenkord et al., 2019; Kostka et al., 2020; Kostka and Hanganu-Opatz, 2023), and, notably, impaired activity in the lateral entorhinal cortex has been found in GE mice (Xu et al., 2021b). In adult mice, inhibition of the lateral entorhinal cortex impairs performance in odor discrimination tasks (Bitzenhofer et al., 2022) and odor-context learning (Persson et al., 2022). This is not to say that odor detection or discrimination happens in the lateral entorhinal cortex, but it shows that this pathway from OB to the hippocampal–prefrontal network is critical for the execution of certain odor-related tasks. Parallel pathways, such as direct projections from the anterior olfactory nucleus and the lateral entorhinal cortex to the PFC (Moberly et al., 2018; Xu et al., 2021b), might provide alternative routes for olfactory information to higher associative areas. However, the pathway through the lateral entorhinal cortex might be particularly vulnerable to reduced OB activity during early postnatal development as indicated by lasting morphological and functional alterations of entorhinal neurons after transient inhibition of OB projection neurons (Chen et al., 2023), as well as in GE mice (Kringel et al., 2023).

For now, we can only speculate how these findings relate to deficits of the olfactory system reported for neuropsychiatric disorders in humans. Olfactory impairment has been suggested as an early indicator for several neuropsychiatric disorders, such as schizophrenia and psychosis (Turetsky et al., 2003; Nguyen et al., 2010; Hasegawa et al., 2022). Patients with first episode psychosis were shown to display deficits in odor identification tasks, and a reduced OB volume and inflammation of the olfactory epithelium have been associated with schizophrenia (Hasegawa et al., 2022; Yang et al., 2024). We found largely normal odor detection and odor learning in GE mice consistent with normal odor-evoked activity during development, but long-term odor memory was impaired. More rigorous behavioral testing might reveal more subtle olfactory dysfunctions early on but the options for behavioral tests in neonatal mice are limited. Alternatively, altered functional maturation of the OB and the feedback projections from higher association areas might accumulate throughout development and only result in olfactory deficits later in life. Future investigations expanding on the present approaches could provide essential insight on how olfactory deficits might act as valuable early diagnostic markers in neuropsychiatric disorders.
